# Targeting Neurotrophins to Specific Populations of Neurons: NGF, BDNF, and NT-3 and Their Relevance for Treatment of Spinal Cord Injury

**DOI:** 10.3390/ijms18030548

**Published:** 2017-03-03

**Authors:** Kathleen M. Keefe, Imran S. Sheikh, George M. Smith

**Affiliations:** Shriners Hospital’s Pediatric Research Center (Center for Neural Repair and Rehabilitation), Lewis Katz School of Medicine at Temple University, 6th Floor Medical Education & Research Building, 3500 N. Broad Street, Philadelphia, PA 19140-4106, USA; tue44530@temple.edu

**Keywords:** spinal cord injury, neurotrophic factors, nerve growth factor, brain-derived neurotrophic factor, neurotrophin-3, neuroprotection, plasticity, regeneration

## Abstract

Neurotrophins are a family of proteins that regulate neuronal survival, synaptic function, and neurotransmitter release, and elicit the plasticity and growth of axons within the adult central and peripheral nervous system. Since the 1950s, these factors have been extensively studied in traumatic injury models. Here we review several members of the classical family of neurotrophins, the receptors they bind to, and their contribution to axonal regeneration and sprouting of sensory and motor pathways after spinal cord injury (SCI). We focus on nerve growth factor (NGF), brain derived neurotrophic factor (BDNF), and neurotrophin-3 (NT-3), and their effects on populations of neurons within diverse spinal tracts. Understanding the cellular targets of neurotrophins and the responsiveness of specific neuronal populations will allow for the most efficient treatment strategies in the injured spinal cord.

## 1. Introduction

In the adult central nervous system, axons fail to regenerate after injury. This lack of regeneration can be attributed to diminished activation of intrinsic growth programs, and a local environment that both lacks growth permissive molecules and contains many growth inhibitory molecules. Even when axons are able to regenerate, they rarely target the correct post-synaptic neurons or form synaptic connections that restore function. An optimal environment for regeneration would include a heightened internal growth state and the presence of molecules that can overcome inhibitory influences to guide axons to appropriate targets, and induce growth only of lesioned neuronal populations, without effecting non-injured populations. In addition, as locomotion and other movement requires multiple motor and sensory pathways for proper integration and re-establishment of movement patterning, growth and connection of multiple neuronal populations may be needed to drive significant functional recovery.

During development, growth-permissive neurotrophic factors allow axons to lengthen and extend towards appropriate targets in the correct numbers. There are currently more than 50 known factors that direct axonal growth and guidance, synapse formation, and pruning of axons and dendrites during development. In the adult, these factors contribute to neuronal survival, axonal plasticity, and synaptic function, including neurotransmitter availability [1,2,3,4,5,6,7,8,9]. However, the expression of many neurotrophic factors is greatly reduced within the adult central nervous system (CNS). Exogenous application of these factors has the potential to create a growth permissive environment after an injury. Here we focus on three factors described as the “classic” neurotrophin family: nerve growth factor (NGF), brain derived neurotrophic factor (BDNF), and neurotrophin-3 (NT-3), and their therapeutic potential for spinal cord injury.

**Figure 1:**
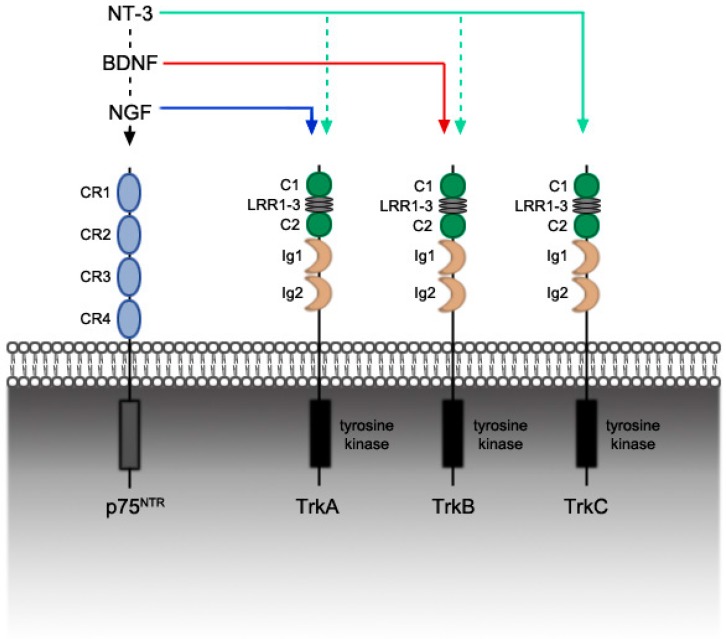
Neurotrophin binding to pan neurotrophin (p75^NTR^) and tropomyosin receptor kinase (Trk) receptors. All neurotrophins bind with low affinity to the p75^NTR^ receptor. Nerve growth factor (NGF) binds with high affinity to TrkA, and brain derived neurotrophic factor (BDNF) with high affinity to TrkB. Neurotrophin-3 (NT-3) binds with high affinity to TrkC, and may bind with low affinity to TrkA or TrkB depending on the cellular context. CR: cysteine-rich repeat, C: cysteine-rich cluster, LRR: leucine-rich repeat, Ig: Immunoglobin-like domain. Solid lines denote high affinity binding, dashed lines denote low affinity binding.

The members of the classical neurotrophin family are structurally similar proteins. They are manufactured as larger, precursor proteins called proneurotrophins, which consist of an N-terminal prodomain and a C-terminal mature domain [10]. The pro-forms, which were once thought only to influence folding of the mature protein, are now recognized as biologically active molecules that may complement or oppose the activity of the mature forms. Mature neurotrophins are created when the pro-forms are cleaved and form non-covalently linked homodimers. Proneurotrophins can either be cleaved intracellularly by furin or proconvertase, and then secreted, or they may be processed extracellularly by plasmin, matrix metalloproteinase-3 (MMP-3), or matrix metalloproteinase-7 (MMP-7) [11,12,13,14,15,16]. These homodimers bind to two main classes of receptors—tropomyosin receptor kinase (Trk) receptors and pan neurotrophin (p75^NTR^) receptors (Figure 1). All members of the family bind with low affinity to the p75^NTR^ receptor. This receptor contains four cysteine-rich repeats (CR1–4). CR2 and CR3 have been implicated as binding sites for neurotrophins [17,18]. The individual neurotrophins bind specifically and with high affinity to Trk receptors, with NGF binding to TrkA, BDNF binding to TrkB, and NT-3 binding to TrkC [19,20,21]. Low affinity NT-3 binding to TrkA and TrkB has also been demonstrated in vitro in neuronal contexts [22,23]. Binding interactions mainly occur in the immunoglobin-like domains (Ig1 and Ig2) of the Trk receptors [21].

Neurotrophin binding to Trk receptors causes receptor dimerization and autophosphorylation of tyrosine residues, ultimately leading to activation of downstream signaling cascades. Several cascades known to be activated or upregulated are phosphatidylinositol 3-kinase (PI3K)/protein kinase B (Akt), Ras/mitogen-activated protein kinase (Ras/MAPK), and phospholipase C-γ (PLC-γ). These pathways have known roles in neuronal survival, axonal outgrowth, and synaptic plasticity [24]. Binding to Trk receptors is influenced by the p75^NTR^ in a number of ways. P75^NTR^ can promote ligand binding to Trk receptors by influencing the confirmation of the receptor, which can increase the affinity of the “preferred” neurotrophin and decrease binding of others [25,26,27]. Several studies have shown that neurotrophins such as NGF require the presence of p75^NTR^ for high affinity binding to TrkA [28]. p75^NTR^ can also promote endocytosis and retrograde transport of neurotrophins to membrane compartments where they can interact with Trk receptors [29,30,31], and may reduce Trk ubiquitination, which can delay the internalization and degradation of the receptors, allowing for longer signaling periods [30]. Additionally, P75^NTR^ can activate pro-survival pathways such as Akt to act synergistically with Trk-mediated neurotrophin effects [32].

Activation of p75^NTR^ receptors can also operate in an antagonistic manner to cell survival and growth, triggering several potential pro-apoptotic cascades. In the absence of Trk, mature neurotrophin binding to p75^NTR^ can activate Jun N-terminal (JNK) kinases, which trigger cell death via activation of p53 [19,33]. JNK activation has specifically been shown to operate in an NGF-dependent manner during p75^NTR^-mediated cell death in cortical oligodendrocytes [34]. In sympathetic neurons, p75^NTR^ can trigger a pro-apoptotic cascade via binding with BDNF through a JNK-independent mechanism [35]. Recent studies of proneurotrophins reveal that these molecules have a higher affinity for the p75^NTR^ receptor than mature forms [14]. p75^NTR^ can form a complex with the sortilin receptor, which binds the N-terminal prodomain of pro-NGF, pro-BDNF, and pro-NT-3. Pro-NGF, which is the most well-defined proneurotrophin, has been implicated in the death of oligodendrocytes, spinal motor neurons, and corticospinal neurons in a p75^NTR^ dependent manner [36,37,38]. Upregulation of this molecule can occur in the injured state [12,13,14]. Thus, the fate of a cell may be determined by the relative abundance of either pro- or mature neurotrophins, or the interplay of Trk and p75^NTR^ receptor availability [33,39].

In the adult uninjured rat, neurons of the spinal cord do not generally produce the neurotrophins discussed in this paper, as evidenced by a lack of detectable mRNA transcript expression [40]. However, the expression of certain Trk receptors is abundant, as mRNA probes show TrkB and TrkC transcripts on the majority of neurons in the gray matter of the spinal cord at all levels examined [40,41,42]. TrkA mRNA was expressed on a small population of neurons scattered throughout the intermediate gray matter [43], and on small diameter nociceptive neurons of the dorsal root ganglia [44]. The presence of the mature form of the Trk receptors can allow for binding and retrograde transport of these factors.

**Figure 2:**
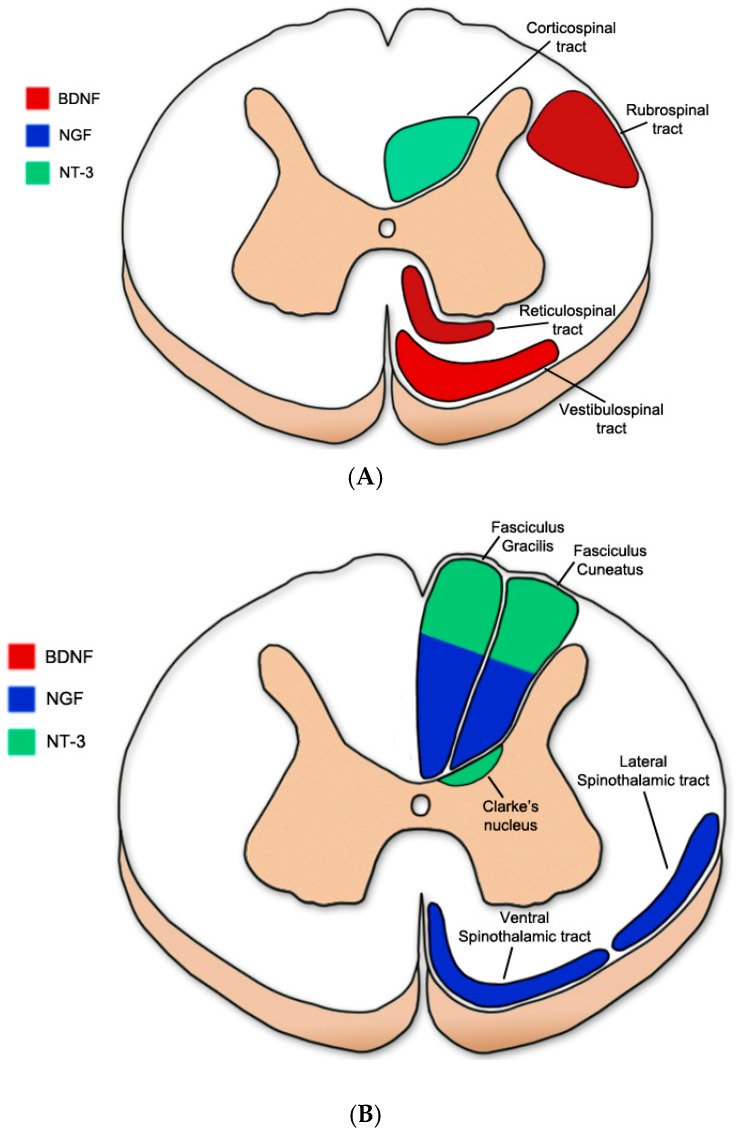
Common motor and sensory tract responsiveness to BDNF, NGF, and NT-3. (**A**) Common motor tracts; (**B**) Common sensory tracts. BDNF: Brain-derived neurotrophic factor, NGF: Nerve growth factor, NT-3: neurotrophin-3.

In the context of spinal cord injury, the presence of Trk receptors may provide treatment targeted to specific spinal cord tracts. Figure 2 outlines neurotrophins which elicit growth responses from common motor (Figure 2A) and sensory (Figure 2B) tracts in the spinal cord (Clarke’s nucleus (proprioceptive): [45]; Corticospinal tract: [46,47]; Fasciculus Cuneatus and Fasciculus Gracilis: [48,49,50,51,52]; Reticulospinal tract: [53,54]; Rubrospinal tract: [54,55,56]; Spinothalamic tract: [50,57,58]; Vestibulospinal tract: [53]).

## 2. Nerve Growth Factor

Nerve growth factor (NGF) was the first neurotrophin discovered in the 1950s by Rita Levi-Montalcini and Viktor Hamburger in mouse sarcoma cultures in vitro [59]. Subsequent studies found a role for NGF in mediating survival and maturation of developing neurons in the peripheral nervous system (PNS). In the central nervous system (CNS), mature NGF has neuroprotective effects and can influence neural responses to injury on cell types that display NGF receptors, such as nociceptive sensory neurons (sympathetic and small diameter peripheral neurons), α motor neurons, and Schwann cells [60,61,62,63,64].

NGF binding with TrkA and p75^NTR^ receptors activates downstream signaling cascades such as MAPK/ERK, PI3K/Akt, and PLC-γ pathways [65,66]. Activation of the MAPK/ERK and PI3K/Akt are both known to promote differentiation and survival of neurons (See Pearson et al., 2001, and Yuan et al., 2003 for reviews) [67,68]. PI3K/Akt phosphorylation of downstream proteins modifies the cell’s cytoskeleton during motility and at the growth cone (See Yuan et al., 2003 for review) [68]. PLC-γ pathways influence the intracellular release of Ca^2+^ by inositol-triphosphate dependent pathways [69]. In turn, this allows for the activation of calcium dependent proteins (e.g., Ca^2+^ calmodulin-dependent protein kinases, Ca^2+^ calmodulin associated targets) and the expression of ion channels and transcription factors [70,71].

The mechanism of NGF-induced axonal sprouting onto sensory neurons is one area of intense study. The expression of NGF within the spinal cord induces robust spouting of nociceptive axons and hyperalgesia [72]. Inhibitors to TrkA or NGF itself can reduce axonal sprouting of nociceptive axons [73,74]. Transplantation of grafts that express and secrete NGF into non-injured and injured rat spinal cords show increased nociceptive axon sprouting [50,58,75,76]. In addition, noradrenergic, cerulospinal, and cholinergic local motor axons sprouting is observed with NGF-expressing transplants [58,76]. NGF is also known to cause severed primary nociceptive sensory pathways to regenerate past the dorsal root entry zone (DREZ) and into the spinal cord [57,77].

The combination of other neurotrophins such as brain derived neurotrophic factor (BDNF), neurotrophin-3 (NT-3), and neurotrophin-4/5 (NT-4/5), or guidance molecules such as semaphorins, with NGF expression alter sprouting of primary sensory pathways in vivo [78,79,80,81,82]. These studies demonstrate improved axonal growth and some functional recovery of nociception [80,81,82] or hindlimb motor function [78,79] in models of spinal cord injury.

As the main trophic effects of NGF are seen in small diameter sensory neurons, clinical trials have focused on issues such as diabetic or HIV-associated neuropathy. Phase II trials using recombinant human NGF (rhNGF) were promising, with improvements seen in sensory components of neurologic examinations and average daily pain assessments [83,84]. However, painful side effects of these trials revealed a dose limit of the rhNGF protein, and a follow-up Phase III trial failed to show any significant ameliorative effect [85]. Whether this was because of the smaller dosage, different study populations, or change in the formulation of the rhNGF was unclear, but its use was abandoned in this context by the manufacturer. A modified rhNGF is currently being evaluated in trials for neurotrophic keratitis by a different company, based on research showing it can promote corneal healing in rats and humans [86].

The nociceptive sprouting encouraged by NGF and accompanying pain reported in both animal models and clinical research has limited the use of this neurotrophin in regenerative studies of spinal cord injury [87,88]. However, research on chronic pain states, a common complaint in patients with SCI, is ongoing. Fasinumab, a drug-based antibody against NGF, found significant improvement in pain scores in the study group [89].

Alternative therapeutic targets to promote neuroprotection could involve inhibiting signaling of proneurotrophins. Levels of pro-NGF are upregulated in the CNS after injury [15,38], and it is believed to be apoptotic when p75^NTR^ receptor levels are high [14,90,91]. Induction of cell death was attributed to pro-NGF in primary superior cervical ganglion cultures, smooth muscle cells, and PC12 cells [14,90,91], and elevated levels of pro-NGF or pro-BDNF are observed in neurological disease states such as Alzheimer’s, autism, or cognitive impairment associated with HIV [15,92,93,94]. Preclinical studies have demonstrated neuroprotection via genetic deletion of sortilin or introduction of pro-NGF specific antagonists in mice [95,96]. NGF antagonists have also demonstrated positive results after spinal cord injury [97], or amyotrophic lateral sclerosis (ALS) [98]. Other treatment options may include modulating the cleavage of pro-forms to encourage the presence of more mature neurotrophins.

## 3. Brain-Derived Neurotrophic Factor

Brain-derived Neurotrophic Factor exerts neuroprotective and growth-promoting effects on a variety of neuronal populations after injury. This is especially apparent in the rubrospinal, reticulospinal, and vestibulospinal tracts, as well as on the proprioceptive neurons of Clarke’s nucleus in the spinal grey matter of the lumbar cord. Neuroprotective outcomes in particular may be attributed to downstream effects of TrkB receptor signaling. Pro-apoptotic molecules such as glycogen synthase kinase 3 (GSK-3), (Bcl-2 associated death promotor) Bad, and JNK are inhibited by TrkB signaling via the PI3-kinase and Akt pathway, allowing cells marked for death to survive. There are also studies showing that BDNF diminishes glutamate-induced apoptotic cell death [99].

BDNF is especially potent when protecting neurons of the rubrospinal tract, whose cells originate in the red nucleus. These neurons can undergo significant atrophy in the weeks following injury [1]. In situ hybridization for TrkB receptor expression found that nearly all rubrospinal neurons express this receptor. Infusion of BDNF via cannula in the vicinity of rubrospinal neurons (RSNs) fully prevented atrophy of these cells. This effect was also seen with NT-4/5, which has a similar binding pattern to TrkB receptors [23,100,101]. A significant increase in the percentage of cells showing mRNA transcripts of growth associated proteins such as GAP-43 and Tα1-tubulin was also found during BDNF application. A follow up study from the same group expressed BDNF via viral vector injection, and found a similar reversal of RSN atrophy and upregulation of regeneration associated genes [102]. These studies focused on acute injury, but RSNs can also be rescued at chronic time points. When a viral vector encoding BDNF was used to transduce these neurons at 18 months post-lesion, increased cell numbers and restoration of neuronal morphology was found in the red nucleus [103].

The neuroprotective effects of BDNF extend to the corticospinal motor system. Lu and colleagues explored both the influence of BDNF on corticospinal motor neuron and axonal growth of corticospinal tract (CST) axons. They found that grafting BDNF-secreting fibroblasts into an aspirated lesion in the cortical area increased survival of motor neurons in the spinal cord, but did not help with the growth of CST axons. Supporting these results, their study found robust TrkB receptor expression on the cell body and dendrites of corticospinal motor neurons, but not on their descending axons [4]. Neuroprotective effects of BDNF were also seen in the primary motor cortex after a T9 spinal cord lesion and grafting of mesenchymal stem cells engineered to secrete BDNF. Quantification of the number of fluorogold-labeled corticospinal neurons showed that BDNF could rescue these neurons when compared to T9 lesioned controls [104].

Studies of BDNF-secreting cells at the site of injury demonstrate that its protective effects can operate over a long distance. This is perhaps most impressively demonstrated by a 2010 study, which found neuroprotective effects in macaque pyramidal neurons when BDNF and NT-3 secreting cells were implanted at a C7 lesion site, a distance of about 10 cm from the cell body. Concurrent studies in rodents from this same group determined that BDNF, and not NT-3, was the factor influencing the survival of these neurons [105].

In addition to neuronal protection, BDNF can enhance regeneration and sprouting of injured axons in the spinal cord [54,55,106,107], or increased remyelination of injured axons [107,108]. Several groups found that application of BDNF induces the upregulation of growth-associated genes such as GAP-43 and T-alpha-1-tubulin in neurons [55,109]. The upregulation of these genes may contribute to enhanced regeneration [110]. It is also thought that TrkB receptor activation of ERK pathway signaling may stimulate regeneration, as ERK can increase levels of cyclic AMP (cAMP), which may be partially responsible for the growth-promoting effects of conditioning lesions in the periphery [110,111]. As TrkB receptors are present on many neurons in the spinal cord, BDNF may act as a general inducer of sprouting and regeneration.

Several groups that reported protection of rubrospinal neurons also found axonal regeneration or regenerative sprouting of the rubrospinal tract [54,55,112,113,114]. A study by Jin and colleagues also noted increased growth of reticulospinal tract and vestibulospinal tract axons into a lesion cavity that contained BDNF-secreting fibroblasts [53]. Growth-promoting effects are also observed with raphespinal [104,113] and coeruleospinal axons when BDNF administration is combined with fetal spinal cord transplants [113].

The effect of BDNF on the growth of corticospinal tract axons is mixed. The Bregman study found increased growth of corticospinal axons when BDNF-soaked gelfoam was applied to a lesion along with fetal spinal cord tissue transplant [113]. The sprouting of CST fibers and partial motor recovery was also seen with BDNF-secreting stem cells, or with transplanted cells and BDNF injected caudal to the lesion [104,106]. However, several other groups failed to see any significant growth of CST axons in response to BDNF [4,115,116], in part perhaps due to the lack of TrkB receptors on these neurons. An interesting study by Hollis and colleagues found overexpressing TrkB on these neurons enhanced axonal expression and allowed CST axons to extend into BDNF-expressing cellular grafts [117].

TrkB is expressed abundantly in neurons of the spinal cord [41,42], potentially making BDNF and other members of the neurotrophin family that bind to it (e.g., NT-4/5) the most universally applicable molecules for injured motor tracts. The abundant expression of this receptor does come with a downside, however, as off-target effects such as pain or spasticity are sometimes associated with BDNF treatment [118,119,120,121]. TrkB is expressed on neurons of the dorsal horn, which receive nociceptive afferents. These neurons can also undergo upregulation of TrkB receptors during injury [122,123]. This may lead to BDNF-facilitated sprouting of C-fibers and increase of synaptic connections in pain pathways, which can then be prevented by blocking BDNF-TrkB signaling [119,120]. Recently, high concentrations of BDNF have been identified to alter chlorine homeostasis in neurons by downregulating the potassium-chlorine cotransporter (KCC2). Under such a condition, the down-regulation of KCC2 results in neuronal hyperexcitability and increased pain and spasticity [124,125,126]. As mentioned previously, another consideration is the relative abundance of the BDNF pro-form, which is upregulated in the injured state [16,127,128], and is associated with neuronal death, neurite collapse, and process retraction in different populations of neurons [127,129,130].

There is some conflicting evidence associated with BDNF treatment and its effects on pain and allodynia. Some authors found reversal of allodynia with BDNF treatment [131,132,133], or that pronociceptive effects may only occur in the non-injured state [134]. There is also speculation that BDNF’s contribution to spasticity may only be induced by continuously high levels of this factor, and thus a regulated or transient dosage may solve this potential problem [135].

Dosage and penetrance are especially important when considering the use of BDNF in a clinical setting. Systemic use of BDNF in clinical trials has been unsuccessful both because of poor tissue penetrance and unfavorable side effects. BDNF crosses the blood-brain barrier only in minimal amounts, making peripheral administration inadequate for treatment, as seen in a large clinical trial seeking to treat amyotrophic lateral sclerosis (ALS) [136]. Methods of delivering BDNF directly to the CNS, such as intraventricular or intrathecal administration, were explored in further ALS trials, along with modification of the protein to increase tissue perfusion. However, BDNF was unable to adequately penetrate brain or spinal cord tissue beyond the superficial layers, failing to reach degenerating motor neurons in the cord [137,138] Currently, methods such as intraparenchymal protein infusion and virus-mediated gene delivery are being explored to increase neurotrophin availability to appropriate neuronal populations [139,140,141,142].

## 4. Neurotrophin-3

The third member of the neurotrophin family, neurotrophin-3 (NT-3), was discovered by a group at the Max Planck Institute in 1990 [143]. This neurotrophin was more challenging to identify than NGF or BDNF because of lower protein abundance. However, researchers used conserved amino acid sequences from NGF and BDNF to create degenerative primers that allowed them to isolate mRNA for a protein with a similar structure and sequence, which they named NT-3. They found that NT-3 supported the survival of chick trigeminal mesencephalic neurons, a group of proprioceptive neurons that innervate skeletal muscle. They also found NT-3 in the liver and visceral organs [143]. This factor was isolated in parallel that same year by another group, who also found NT-3 in the kidney, lung, cerebellum, medulla, and hippocampus, and suggested that the broad distribution of this factor spoke to its role as a trophic factor for growing sympathetic and sensory neurons [144].

Later work found NT-3 to be important for the survival of several other groups of neurons. Studies in NT-3 null mutants found the developmental lack of a TrkC-expressing subpopulation of dorsal root ganglionneurons [145], as well as the elimination of muscle spindle 1a proprioceptive neurons [146]. Both studies resulted in abnormal movement patterns and very limited survival. In vitro, NT-3 also contributes to the survival of neurons of the hippocampus, sympathetic ganglia, dorsal root ganglia, and dopaminergic and GABAergic neurons of the ventral mesencephalon [60,147].

NT-3 is the most indiscriminate binding partner of the three mentioned neurotrophins, binding with lower affinity to TrkA, TrkB, and the p75^NTR^ receptor, and to the TrkC receptor with the highest affinity [148,149]. Thus far, NT-3 is the only neurotrophin isolated that binds with high affinity to the TrkC receptor. This is important because neurons of the corticospinal tract express the TrkC receptor [150]. The CST is the largest, and one of the most important, motor tracts that descend from the brain. It is involved in fine motor skills such as detailed hand and digit function. In the developing spinal cord, high levels of the TrkC receptor are found in the deep layers of the developing cortex, where CST neurons originate [151]. NT-3 mRNA is highly expressed in the developing spinal cord in motor neurons. Local injection of NT-3 into projected CST targets results in increased collateral sprouting of the CST, which is necessary for target finding, innervation, and synapse formation [47,152]. Though NT-3 expression is minimal in the adult spinal cord, the abundant expression of TrkC allows the responsiveness of the CST to mimic some of the observations seen during development. Grafts of cells engineered to express NT-3 into lesion sites allow for CST growth over short distances both at acute [46,153,154] and chronic time points [155]. These studies also find a modest amount of functional recovery in either grid walking skills or locomotor scores.

Other studies using viral vector mediated delivery of NT-3 found regrowth of CST fibers when combined with nerve implants [156], or when injected into the rostral spinal cord [157], or the triceps muscle [158]. Collateral sprouting of CST axons can also be seen across the midline towards denervated motor neurons that express NT-3 [159,160]. CST axons seem to be the major benefactor of this neurotrophin, as a lack of sprouting has been found in other tracts, such as rubrospinal or cerulospinal [46].

Another advantage of NT-3 is that it is not associated with off-target effects such as pain or spasticity. In fact, NT-3 is currently being investigated in clinical trials as a treatment for peripheral neuropathies, which are often associated with chronic pain and allodynia. These trials are based on evidence that NT-3 prevents degeneration of peripheral sensory axons and can improve functional response in these neurons [161,162,163]. Significant improvement in the Neuropathy Impairment Score was seen in the NT-3 study group in one trial, and an increase in the number of small myelinated fibers assessed from sural nerve biopsies after treatment [163]. Subcutaneous administration of NT-3 is also being considered in Charcot-Marie Tooth neuropathy, as supported by animal studies [164].

## 5. Conclusions

The positive effects of neurotrophins on the growth, survival, and guidance of injured neurons of the spinal cord make them promising candidates for inclusion in treatment strategies. Thorough knowledge of their effects on specific populations of neurons will allow for the most efficient targeting, and awareness of receptor interaction can allow for fine tuning of dosage and avoidance of off-target effects. In combination with treatments that help ameliorate lesion environments such as stem cell grafts or nerve bridges, these factors have the potential to facilitate meaningful recovery after spinal cord injury.

## Abbreviations

BDNF	Brain-derived Neurotrophic Factor
NGF	Nerve Growth Factor
NT-3	Neurotrophin-3
CST	Corticospinal Tract
